# Iatrogenic ischiofemoral impingement due to high tibial osteotomy with overvalgization: a case report

**DOI:** 10.1186/s13256-022-03257-2

**Published:** 2022-02-04

**Authors:** Christian Konrads, Sufian S. Ahmad, Tina Histing, Maher Ibrahim

**Affiliations:** 1 Department for Trauma and Reconstructive Surgery, BG Klinik, University of Tübingen, Schnarrenbergstr. 95, 72076 Tübingen, Germany; 2 Center for Musculoskeletal Surgery, Charité – University Medical Center Berlin, Berlin, Germany; 3 Department of Orthopaedic Surgery, Nyon Hospital, Nyon, Switzerland

**Keywords:** Hip, Knee, Malalignment, Realignment, Varus, Valgus

## Abstract

**Background:**

Open wedge high tibial osteotomy is a standard procedure for frontal realignment. It is indicated in varus knee with reduced mechanical medial proximal tibia angle. Overcorrection producing a mechanical medial proximal tibia angle out of the normal range (85–90°) is not recommended because this would lead to unphysiological joint-line orientation. Osteotomies around the knee also influence the adjacent ankle and hip joints. For the hip, it is known that frontal alignment of the leg influences the ischiofemoral space. A decreased ischiofemoral space can lead to painful impingement between the ischial bone and the lesser trochanter.

**Case presentation:**

A 53-year-old German woman presented with severe ischiofemoral impingement symptoms and valgus malalignment of the left leg after open wedge high tibial osteotomy, which was indicated and performed by an orthopedic surgeon with intention to treat medial knee pain due to degenerative arthritis of the medial compartment years after medial meniscectomy. The mechanical medial proximal tibia angle was 100.5°. We performed closed wedge high tibial osteotomy producing a mechanical medial proximal tibia angle of 90.0° and normal joint-line orientation. The hip pain was gone immediately after the surgery, and the patient had no signs of ischiofemoral impingement or hip pain at last follow-up 12 months after closed wedge high tibial osteotomy.

**Conclusions:**

Frontal realignment osteotomy around the knee can create problems at adjacent joints. Overvalgization of the proximal tibia made the patient compensate by hyperadduction of the hip to enable full foot sole contact with the floor. Hyperadduction of the hip decreased the ischiofemoral space, leading to severe impingement. Therefore, meticulous planning of osteotomies is important not to produce unphysiological situations or unwanted negative effects at the level of an adjacent joint.

## Background

Open wedge high tibial osteotomy (owHTO) is a standard procedure for frontal realignment [1, 2]. It is indicated in varus knee with reduced mechanical medial proximal tibia angle (mMPTA) [3]. Overcorrection producing a mMPTA out of the normal range (85–90°) is not recommended because this would lead to unphysiological joint-line orientation [4, 5].

Osteotomies around the knee also influence the adjacent joints—ankle and hip [6–9]. For the hip, it was demonstrated earlier that frontal alignment of the leg influences the ischiofemoral space [9], but this has not become common knowledge yet. A decreased ischiofemoral space can lead to impingement between the ischial bone and the lesser trochanter [7, 9]. This is demonstrated and illustrated by the present case.

Unphysiological valgus knee alignment is normally compensated by the patient by adducting the hip to enable full foot sole contact with the floor [9]. As hip adduction decreases the space between the ischial bone of the pelvis and the lesser trochanter of the femur, ischiofemoral impingement can result [9]. This is known to be an important extraarticular source of hip pain.

## Case presentation

A 53-year-old German woman presented with severe ischiofemoral impingement symptoms and valgus malalignment of the left leg after owHTO, which was indicated and performed by an orthopedic surgeon with intention to treat medial knee pain due to degenerative arthritis of the medial compartment years after medial meniscectomy. The initial osteotomy was performed 4 years previously, and the patient has been severely symptomatic since this surgery.

**Fig. 1:**
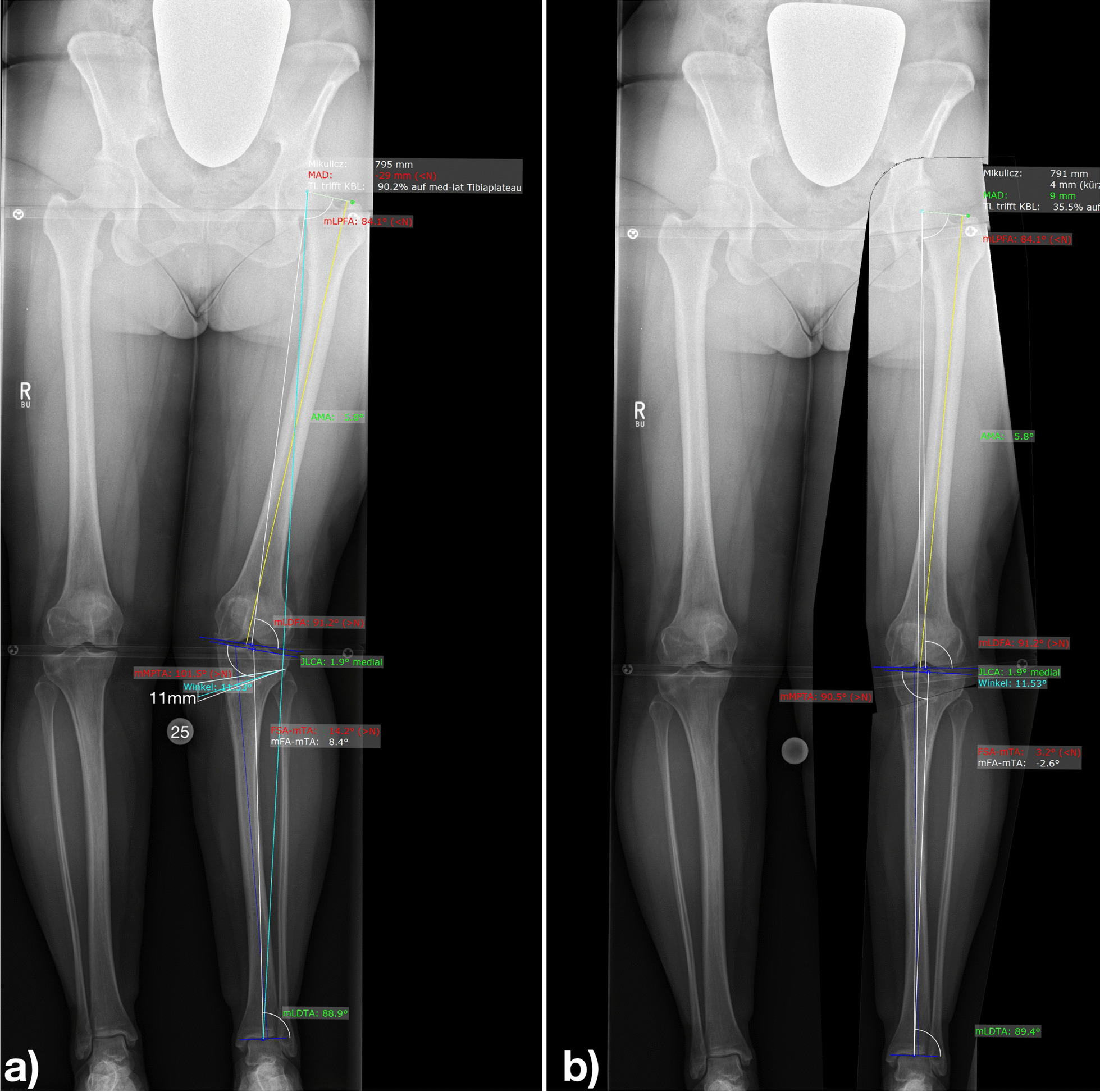
Long-leg standing radiograph. **a** Valgus aligned knee with oblique joint-line and minimal ischiofemoral space between the ischial bone and the lesser trochanter. Using mediCAD software, a medial closed wedge high tibial osteotomy for varization is planned on the native X-ray showing the planned surgical procedure (11-mm closed wedge high tibial osteotomy) and **b** the planned result after osteotomy

**Fig. 2:**
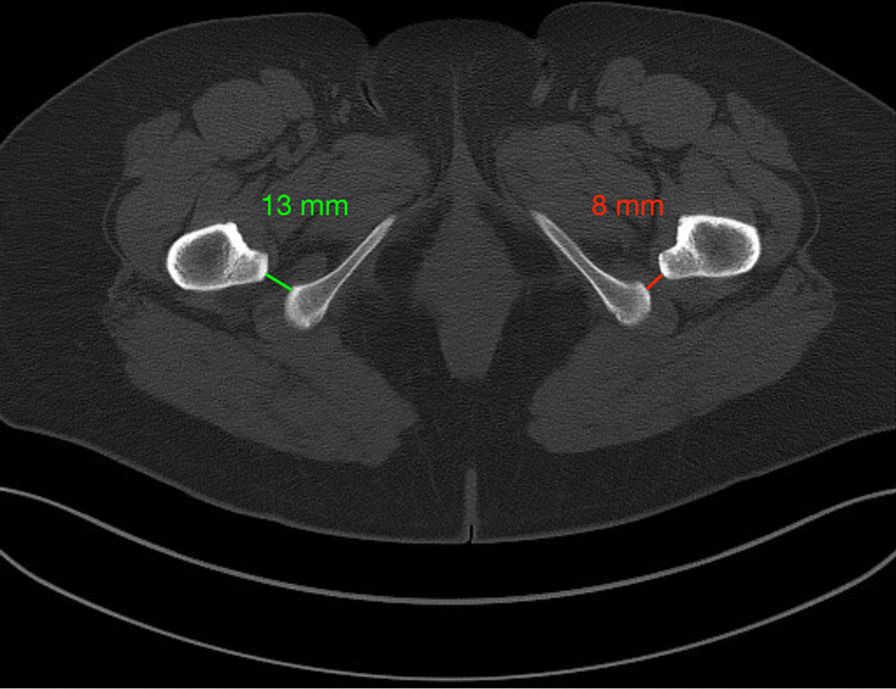
Axial computed tomography slide at level of lesser trochanters. The ischiofemoral distance is smaller on the left than right side

On clinical examination, the Faber test was positive and external rotation of the hip in extension was painful, too. Radiologically, the mMPTA was 101.5° (Fig. 1a). The patient almost could not walk because she needed to adduct her left hip so much to enable her foot to approach the floor evenly. With a positive Faber test, psoas sign, and posterior impingement test, the range of motion (ROM) of the affected hip was Ext/Flex 0/0/120°, Abd/Add 45/0/0°, Aro/Iro 40/0/15°. The WOMAC score was 20.3. The Merle D’Aubigné and Postel score was 13. Torsional malalignment was excluded by torsional CT analysis. On axial CT slides, a reduced ischiofemoral distance was found on the affected left side (Fig. 2).

**Fig. 3:**
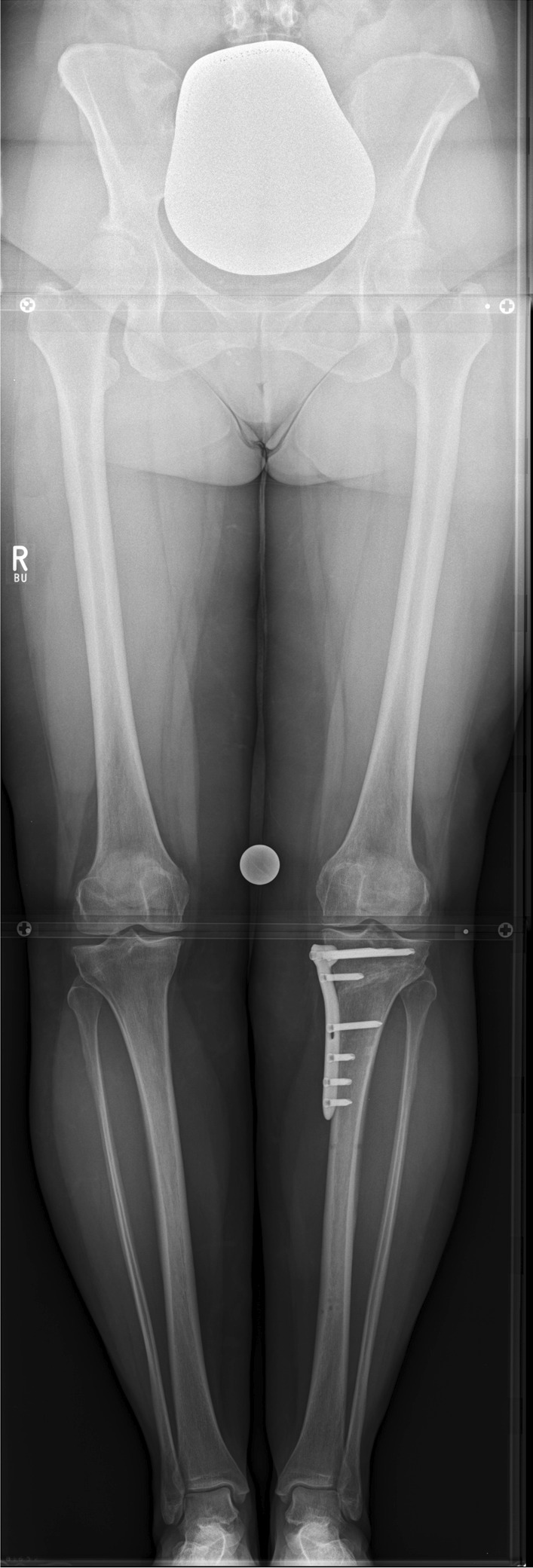
Long-leg standing radiograph showing normal knee alignment and increased ischiofemoral space postoperatively (after closed wedge high tibial osteotomy)

We planned (Fig. 1a, b) and performed cwHTO producing a mMPTA of 90.0° and normal joint-line orientation (Fig. 3). The hip pain was gone immediately after surgery, and the patient had no signs of ischiofemoral impingement or hip pain at the last follow-up 12 months after cwHTO. At this time, the ROM was Ext/Flex 10/0/120°, Abd/Add 45/0/25°, Aro/Iro 45/0/20°. The WOMAC score was 87.5, and the Merle D’Aubigné and Postel score was 18.

## Discussion and conclusions

In the presented case of a 53-year-old patient, massive overvalgization of about 10° deteriorated the function of the lower extremity tremendously. In varus situations of the knee due to medial joint space narrowing without bony malalignment per se, one should be cautious when performing realignment osteotomies. In these cases, medial unicompartmental knee arthroplasty might be more appropriate.

For sufficient preoperative planning, it is recommended to at least analyze the long-leg axis, the joint-line convergence angle (JLCA), and the following bony angles around the knee on a standardized long-leg standing radiograph: mechanical lateral distal femur angle (mLDFA) and mMPTA [10–12]. Figure 1 shows the computer-aided digital planning, which was conducted preoperatively.

Slight overvalgization is accepted in cases with medial gonarthritis, but no unphysiological bony angles or oblique JLCA should be created. To avoid this, often double-level osteotomy of the proximal tibia and the distal femur is indicated in cases with bony malalignment [4, 13, 14]. It was demonstrated earlier that joint-line obliquity (JLO) of more than 3° is associated with worse clinical outcomes after medial owHTO [15]. Overcorrection of more than 3° should be avoided [15].

In conclusion, frontal realignment osteotomy around the knee can create problems at the hip. Overvalgization of the proximal tibia made the patient compensate by hyperadduction of the hip to enable full foot sole contact with the floor. Hyperadduction of the hip decreased the ischiofemoral space, leading to severely painful symptoms of ischiofemoral impingement. Therefore, meticulous planning of osteotomies according to the described principles is important to avoid unphysiological situations or unwanted negative effects at the knee or the level of an adjacent joint.

## Data Availability

The datasets used and analyzed are available from the corresponding author on reasonable request.

## Abbreviations

CT: Computed tomography
cw: Closed wedge
HTO: High tibial osteotomy
JLCA: Joint-line convergence angle
JLO: Joint-line obliquity
mLDFA: Mechanical lateral distal femur angle
mMPTA: Mechanical medial proximal tibia angle
ow: Open wedge
ROM: Range of motion
WOMAC: Western Ontario and McMaster Universities Osteoarthritis
